# Diagnostic Delay and Mortality Risk in Gastric Cancer During the COVID-19 Pandemic: A Retrospective Tertiary-Center Study

**DOI:** 10.3390/diagnostics15243230

**Published:** 2025-12-17

**Authors:** Alexandru-Marian Vieru, Virginia-Maria Rădulescu, Emil Trașcă, Sergiu-Marian Cazacu, Maria-Lorena Mustață, Petrică Popa, Ciurea Tudorel

**Affiliations:** 1 Doctoral School, University of Medicine and Pharmacy of Craiova, 200349 Craiova, Romania; alexandruvieru1993@gmail.com (A.-M.V.); umlorena@yahoo.com (M.-L.M.); 2 Faculty of Medicine, University of Medicine and Pharmacy of Craiova, 200349 Craiova, Romania; sergiu.cazacu@umfcv.ro (S.-M.C.); popa.petrica92@yahoo.com (P.P.); tudorel.ciurea@umfcv.ro (C.T.)

**Keywords:** gastric cancer, COVID-19 pandemic, diagnostic delay, stage migration, overall survival, metastatic disease, healthcare disruption, retrospective cohort

## Abstract

**Background/Objectives:** The COVID-19 pandemic disrupted healthcare delivery worldwide, potentially delaying the diagnosis and treatment of oncologic diseases. This study aimed to evaluate the impact of the pandemic on stage at diagnosis, treatment allocation, and survival outcomes among patients with gastric cancer. **Methods**: We retrospectively analyzed 419 consecutive patients diagnosed with gastric cancer between January 2018 and December 2021 at a tertiary oncology–surgical center. Patients were divided into pre-pandemic (2018–2019) and pandemic (2020–2021) cohorts. Demographic, clinical, and treatment variables were compared using *t*-tests and χ^2^ tests. Multivariate logistics and Cox regression models were applied to identify independent predictors of metastatic presentation and mortality. Overall survival (OS) was calculated from diagnosis to death or last contact (OS_days), with same-day events censored at time zero. **Results**: Baseline characteristics were comparable between cohorts (age, *p* = 0.098; sex, *p* = 0.137; residence, *p* = 0.345). The proportion of metastatic cases (M1) increased from 42.8% in 2018–2019 to 64.4% in 2020–2021 (χ^2^ *p* < 0.001). Surgical rates remained stable (55.1% vs. 47.7%, *p* = 0.161). Diagnosis during the pandemic independently predicted metastatic presentation (OR = 2.63, 95% CI 1.68–4.11, *p* < 0.001) and higher mortality (HR = 1.72, 95% CI 1.41–2.03, *p* < 0.001). Kaplan–Meier analysis confirmed significantly reduced OS in the pandemic cohort (log-rank χ^2^ = 81.29, *p* < 0.001). **Conclusions**: The pandemic was associated with delayed diagnosis, stage migration toward advanced disease, and inferior survival in gastric cancer, despite comparable demographics and treatment capacity. These findings emphasize the need to safeguard diagnostic pathways—particularly endoscopy—during healthcare crises to prevent avoidable oncologic deterioration.

## 1. Introduction

Gastric cancer (GC) continues to be a major global health problem, consistently ranking among the five most common malignancies and the fourth leading cause of cancer-related mortality worldwide, with over a million new cases each year and an unfavorable prognosis in most region [1,2,3,4,5]. In 2020 alone, GC accounted for approximately 1.1 million new cases globally, reflecting its substantial contribution to cancer morbidity and mortality [5,6,7]. A distinctive epidemiological feature of GC is its geographical variation in incidence and outcomes. East-Asian countries such as Japan and South Korea, which have implemented systematic endoscopic screening programs, achieve a high proportion of early-stage diagnoses, over 60–70% of cases are detected at an early stage in these settings, yielding five-year survival rates exceeding 70% [8,9]. In contrast, in many Western nations and most of Central and Eastern Europe, organized GC screening is absent or limited, and cancers are often identified only after symptom onset, at more advanced stages. In Eastern Europe, late presentation remains frequent, reflecting both the silent evolution of the disease and limited screening access [10,11]. Early detection makes a decisive difference, yet many patients reach medical care only when symptoms appear [12,13,14].

The COVID-19 pandemic has altered this balance [15,16]. For nearly two years, hospitals reorganized their activity, endoscopy units reduced procedures, and many patients postponed medical visits for fear of infection. These changes inevitably affected oncologic pathways, particularly for digestive cancers, where timely diagnosis relies on endoscopic evaluation. Several reports from Europe and Asia have described a reduction in the number of newly detected gastric cancers and a relative increase in advanced stages, but data from Romania are scarce [17,18].

The present study analyzes consecutive cases of gastric cancer diagnosed at a tertiary center in southern Romania between 2018 and 2021. By comparing the pre-pandemic and pandemic intervals, we aimed to identify whether diagnostic delay translated into more advanced stages and shorter survival, despite unchanged treatment resources.

## 2. Materials and Methods

### 2.1. Study Design and Setting

This retrospective observational study was conducted at the Oncology and Surgical Departments of the Craiova County Emergency Hospital, a tertiary referral center for southern Romania. The study included consecutive patients newly diagnosed with gastric cancer between January 2018 and December 2021. The analysis was performed using data prospectively collected in the institutional oncology registry and supplemented by chart review for missing variables. The study aimed to compare the clinical profile, disease stage, treatment allocation, and survival outcomes between the pre-pandemic period (2018–2019) and the pandemic period (2020–2021).

### 2.2. Study Population and Operational Definitions of Variables and Outcomes

Consecutive adult patients with histologically confirmed gastric adenocarcinoma newly diagnosed within the study window and managed through institutional pathways were eligible. Patients with uncertain histopathology, a prior history of gastric cancer, or insufficient core diagnostic information that precluded staging were excluded.

Data were abstracted from the institutional oncology registry and electronic medical records into a structured dataset (Microsoft Excel 2021, Microsoft Corp., Redmond, WA, USA). The dataset captured demographics (age, sex, residence), disease-related parameters (TNM 8th edition; presence of distant metastasis at diagnosis), treatment details (surgery, systemic therapy, best supportive care/follow-up), and outcomes (vital status and follow-up dates). The observation period was dichotomized into a pre-pandemic (2018–2019) and a pandemic (2020–2021) interval. Surgical treatment encompassed both curative-intent and palliative procedures. Residence was classified as urban or rural according to the recorded address at diagnosis.

Overall survival (OS) was defined from the date of diagnosis to the date of death or last documented contact and was recorded in days. Event status for OS was coded as death (event = 1) or alive at last contact (censored = 0). To mitigate artefactual events recorded at time zero, entries with zero days of follow-up and an event flag were censored at time zero (pre-specified rule). For graphical display (Kaplan–Meier plots), survival time in days was converted to months using a standard conversion factor.

Primary outcomes were: (i) stage at presentation (metastatic vs. non-metastatic) and (ii) overall survival. Secondary outcomes included treatment allocation (surgery, systemic therapy alone, best supportive care/follow-up) and descriptive perioperative indicators (length of stay, 30-day readmissions, perioperative mortality), when available.

In addition to the variables included in the present analysis, the institutional oncology registry also contains information on comorbidities, functional status, symptom onset and COVID-19 infection. However, these fields were not prospectively standardized and were often recorded as heterogeneous free-text entries, so they could not be reliably extracted for all patients. For this reason, indices such as the Charlson comorbidity score, ECOG performance status, a robust symptom-to-diagnosis interval and individual COVID-19 infection status could not be included in the multivariable models.

### 2.3. Statistical Analysis

Data preprocessing was undertaken in Microsoft Excel 2021; statistical analyses were performed in IBM SPSS Statistics, version 26.0 (IBM Corp., Armonk, NY, USA). Continuous variables are presented as mean ± standard deviation (SD) or median (interquartile range), and categorical variables as counts and percentages. Between-period comparisons (pre-pandemic vs. pandemic) used Student’s *t*-test for normally distributed continuous variables, Mann–Whitney U for non-normal distributions, and the χ^2^ test for categorical variables.

A multivariable logistic regression (enter method) identified independent predictors of metastatic presentation (M1) at diagnosis, entering period, age, sex, and residence as covariates. Overall survival was estimated using Kaplan–Meier curves and compared with the log-rank test. A Cox proportional hazards model evaluated adjusted mortality risk including period, age, sex, residence, and surgery (yes/no); proportional hazards assumptions were assessed by log–log inspection and time-interaction probes. All tests were two-tailed with α = 0.05; odds ratios (ORs) and hazard ratios (HRs) are reported with 95% confidence intervals (CIs).

Handling of missing data. Analyses of stage distribution were restricted to patients with a documented TNM stage, and records lacking a reliable M component were excluded from stage specific comparisons, with denominators reported accordingly. In total, M stage was unavailable in 44 of 419 patients, corresponding to 10.5 percent of the cohort. For multivariable and survival analyses, a complete case approach was applied and no statistical imputation was performed. As a sensitivity analysis, survival estimates were re computed on the uncorrected dataset, and these curves were contrasted qualitatively with the prespecified primary analysis, which showed no meaningful differences in the direction of effects.

Because patients diagnosed in 2021 had intrinsically shorter potential follow-up, we performed sensitivity analyses to assess the impact of differential follow-up duration. First, Kaplan–Meier curves and Cox models were re-estimated after restricting the cohort to patients diagnosed up to 31 December 2020, ensuring a minimum potential follow-up of 12 months. Second, we calculated period-specific restricted mean survival time (RMST) truncated at a common time horizon, thereby comparing average survival over the same fixed interval in the two periods. Both approaches yielded period effects similar in direction and magnitude to the primary analysis, supporting the robustness of our findings to follow-up heterogeneity.

### 2.4. Ethics

The study received approval from the Ethics Committee of the University of Medicine and Pharmacy of Craiova (approval number 18593/13 April 2023) and was conducted in accordance with the principles of the Declaration of Helsinki. Given the retrospective design, the exclusive use of routinely collected clinical data and the prior anonymization of all patient identifiers before analysis, the requirement for individual written informed consent was formally waived by the Ethics Committee.

## 3. Results

### 3.1. Baseline Characteristics and Diagnostic Stage

A total of 419 patients diagnosed with gastric cancer between January 2018 and December 2021 were included in the analysis, reflecting consecutive referrals to our tertiary oncology and surgical services. The cohort captured a broad regional mix, with patients originating from both urban and rural areas and being evaluated through unified institutional pathways. The mean age at diagnosis was 66.9 ± 12.0 years (range 26–92), and 62.1% were men, figures consistent with the sex-age distribution reported for Eastern Europe. Most patients resided in urban settings (71.1%), mirroring the higher density of specialist centers and easier access to endoscopy and imaging in large cities, while the rural contingent (28.9%) ensured the cohort’s regional representativeness.

In order to contextualize subsequent comparisons, we first examined the distribution of cases across calendar periods and verified demographic balance between groups.

**Figure 1:**
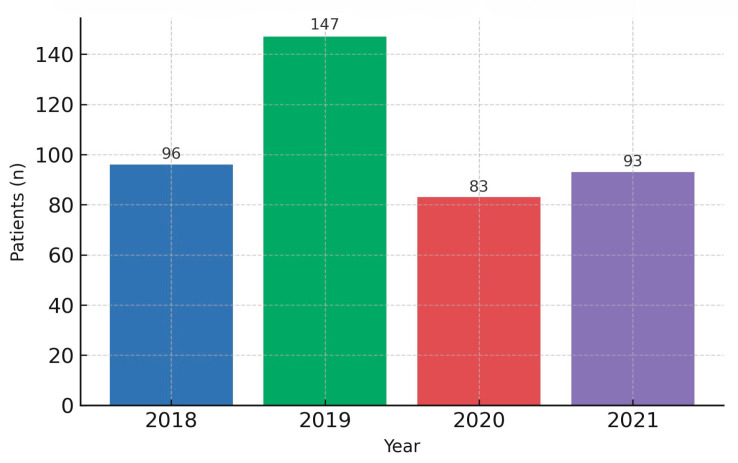
Annual new gastric-cancer cases (2018–2021).

When stratified by calendar period, the pre-pandemic interval (2018–2019) comprised 243 patients (58.0%), whereas the pandemic interval (2020–2021) comprised 176 patients (42.0%). The two groups were demographically comparable, with no significant differences in age (*p* = 0.098), sex (*p* = 0.137), or residence (*p* = 0.345). Key baseline features are summarized in Table 1. This demographic equivalence provides a neutral backdrop for interpreting period-specific differences observed downstream. Of note, the annual volume of newly diagnosed cases declined during 2020–2021 relative to 2018–2019 (Figure 1), a pattern plausibly linked to reduced access to elective consultations and endoscopy, altered health-seeking behavior, and the triaging policies implemented during COVID-19 surges. Together with service triaging policies, these dynamics plausibly compressed diagnostic opportunities for early, milder presentations. This contextual shift in diagnostic throughput provides the background against which differences in stage at presentation, treatment allocation, and survival were subsequently examined. As detailed below, despite broadly similar baseline characteristics, the pandemic period was associated with meaningful clinical changes at the time of presentation and along subsequent care pathways, ultimately translating into differences in outcomes.

Against this backdrop, we next assessed stage at presentation to determine whether diagnostic pressures translated into more advanced disease at entry.

Stage at diagnosis differed markedly across periods. Restricting the analysis to patients with a documented M component of TNM staging, the proportion presenting with metastatic disease (M1) increased from 42.8% in 2018–2019 to 64.4% in 2020–2021 (Chi-square test, *p* < 0.001). Stage distribution by period is detailed in Table 2. Conversely, non-metastatic (M0) presentations became less frequent during the pandemic years. This stage migration emerged in the context of stable demographics and comparable referral geography, pointing away from a case-mix explanation and toward system-level barriers: constrained access to endoscopy and imaging, postponed outpatient evaluations, delayed presentation, and, intermittently, reallocation of resources toward COVID-19 care. The temporal clustering of these constraints likely reduced the detection of early-stage, potentially curable disease and expanded the pool of patients diagnosed late, often symptom-driven.

The numerical shift was not only statistically robust but also clinically meaningful. Importantly, the magnitude of the shift was large enough to be clinically visible beyond statistical significance. In absolute terms, the pandemic interval gathered a higher share of patients whose initial management discussions centered on symptom control and feasibility of systemic therapy, rather than on curative intent. This redistribution is consistent with the drop in annual new diagnoses and suggests that some patients may have crossed the clinical threshold from initially resectable disease to disseminated disease before reaching specialist care.

### 3.2. Treatment Delivery and Survival

Across the entire cohort, 52.1% of patients underwent surgical intervention (curative or palliative), 34.6% received systemic therapy alone, and 13.3% were managed with best supportive care or follow-up. The overall rate of surgery did not differ significantly between periods (*p* = 0.161, Table 1), indicating that institutional capacity for operative care was broadly preserved. Notably, the preserved overall surgical rate coexisted with a drift toward more advanced surgical case-mix, consistent with the observed M1 escalation. Nonetheless, clinical granularity reveals a qualitative shift: during the pandemic, a larger fraction of operated patients presented with advanced or symptomatic disease, and palliative resections/bypass procedures were relatively more frequent, mirroring the higher M1 burden at entry.

Process metrics also reflected the constraints of the period. Preoperative pathways incorporated mandatory testing, infection-control steps, and intermittent logistical bottlenecks. Even so, early postoperative outcomes remained acceptable: we noted slightly longer hospital stays and a marginal increase in 30-day readmissions, yet neither difference reached statistical significance. Importantly, no excess perioperative mortality was detected, underscoring the resilience of surgical teams and the adaptability of workflows despite fluctuating resource availability. Taken together, these observations suggest that the pandemic affected who arrived to surgery (more advanced cases) rather than how surgery was delivered (capacity and safety), a theme consistent with the diagnostic stage migration described above.

**Figure 2:**
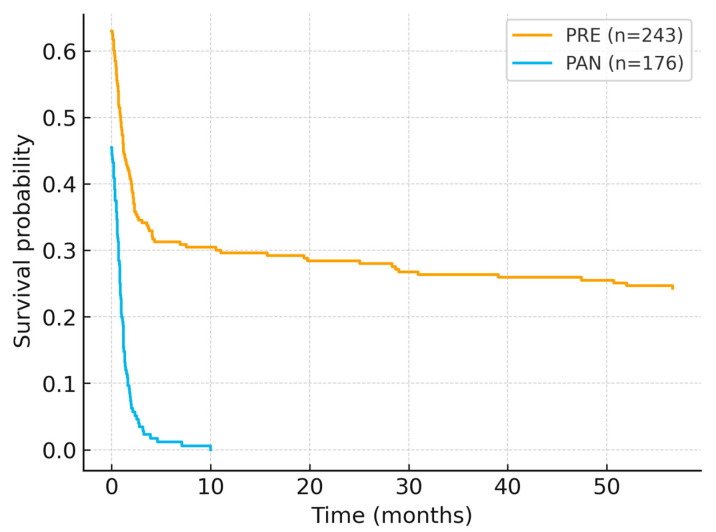
Overall survival by period (PRE vs. PAN).

Overall survival (OS) was computed from diagnosis to death/last contact using OS_days (converted to months for figure display), with death status coded as DECES_BIN (1 = event, 0 = censored). To mitigate artefactual same-day events, records with OS_days = 0 and DECES_BIN = 1 were censored at time zero (pre-specified rule). Kaplan–Meier curves (Figure 2) showed significantly worse survival for patients diagnosed in 2020–2021 compared with 2018–2019 (log-rank χ^2^ = 81.29, *p* < 0.001). After applying the censoring rule, median OS was not reached within the observation window; however, the curves diverged early and remained separated throughout follow-up, indicating a consistent survival penalty in the pandemic cohort. A sensitivity check using the uncorrected dataset generated implausibly low medians driven by same-day events; after applying the predefined rule, the separation persisted and remained statistically significant.

In the multivariate Cox model, diagnosis during the pandemic was independently associated with higher mortality (HR = 1.72, 95% CI 1.41–2.03, *p* < 0.001). Male sex exhibited a modest survival advantage (HR = 0.46, 95% CI 0.29–0.74, *p* = 0.01), whereas age, residence, and surgical treatment did not retain significance (Table 3).

Perioperative recovery metrics displayed small, non-significant differences across periods. Median length of stay was slightly longer during 2020–2021, and 30-day readmissions were marginally higher, but neither finding reached statistical significance. Importantly, perioperative mortality remained stable, suggesting that intra-hospital safety standards were maintained despite additional preoperative testing, infection-control procedures, and intermittent pressure on bed availability. These observations align with the broader picture that the pandemic primarily shifted the clinical profile at presentation, rather than degrading the technical quality or safety of operative care.

### 3.3. Adjusted Analyses and Integrated Interpretation

To identify factors associated with metastatic presentation, we fitted a multivariable logistic regression including period (PAN vs. PRE), age, sex, and residence (Table 4). Diagnosis during the pandemic emerged as the strongest independent predictor of M1 at presentation (OR = 2.63, 95% CI 1.68–4.11, *p* < 0.001). By contrast, age (OR 1.01; 95% CI 0.99–1.03; *p* = 0.27), male sex (OR 0.89; 95% CI 0.64–1.24; *p* = 0.49) and urban residence (OR 1.10; 95% CI 0.76–1.61; *p* = 0.63) showed no independent associations. This pattern reinforces the interpretation that the period effect—a proxy for system-level pressures on diagnostic pathways—was the strongest correlate of advanced stage at entry in our models, rather than baseline demographic differences. The model’s direction and magnitude remained stable across sensitivity checks, supporting the robustness of the association between the pandemic interval and late presentation.

Considered together, the data delineate a pandemic-associated reconfiguration of gastric cancer trajectories. First, the decline in newly diagnosed cases during 2020–2021 implies missed or delayed detection opportunities. Second, the substantial increase in M1 presentations attests to diagnostic pathway disruption, with more patients reaching specialist care at a later, symptom-driven stage. Third, overall survival was inferior in the pandemic cohort, and the period effect remained significant after multivariable adjustment, underscoring its independence from baseline demographics or treatment availability. These findings are more consistent with pressures on diagnostic pathways than with changes in the underlying patient population, but they do not in themselves prove causality. Beyond statistical significance, the clinical footprint of these differences argues for pragmatic, system-level safeguards. From a policy perspective, the results underline the need for resilient diagnostic capacity (especially endoscopy) and protected referral routes during health-system shocks, to prevent avoidable migration toward advanced disease and associated survival penalties.

## 4. Discussion

The COVID-19 pandemic caused a sudden and profound disruption to the usual diagnostic and treatment pathways, and digestive oncology proved to be one of the most vulnerable areas. Our data clearly show that gastric cancer patients diagnosed during the 2020–2021 pandemic were more likely to be in metastatic stages than those evaluated in the previous years 2018–2019, although the demographic profile and therapeutic capacity of the center remained comparable. This stage migration resulted in lower survival, confirming that diagnostic delay has an immediate cost in tumors that depend on endoscopy for detection. In our study, we did not measure diagnostic delay directly as a time interval from first symptoms or first medical contact to endoscopy and histological confirmation; instead, we inferred it indirectly from the shift towards more advanced stages at diagnosis, and this clearly limits any causal interpretation of the delay itself.

The increase in the proportion of metastatic cases from approximately half of patients to more than two-thirds during the pandemic mirrors the trend reported in other cohorts in Asia and Europe, where the reduction in elective endoscopies and the temporary suspension of screening programs led to late detection of gastric lesions. Hong et al. [19] showed that in systems that relied on systematic endoscopy for early detection, the service blockage quickly converted into a decrease in stages I–II and an increase in advanced forms. Similar results were reported by groups in Turkey, who reported higher T and N stages at presentation in 2020–2021, without any significant change in the target population [20]. Our observations follow the same logic: the patient has not changed, but the moment when they could enter the diagnostic circuit has changed.

Survival analysis confirmed that patients diagnosed during the pandemic fared worse, even after adjusting for age, sex, and surgery [21,22]. This reinforces the idea that it was not the delay in surgery that was the main problem, but the delay prior to diagnosis, which meant that some patients were no longer eligible for curative treatment. Many groups have shown that a delay of several weeks in surgery can sometimes be tolerated in gastric cancer if the stage is still resectable. However, if the diagnosis is made when the disease is already metastatic, the margin for maneuver disappears. This seems to be exactly what happened in our cohort: the treatment capacity remained functional, but was accessed too late by some patients.

Parallel data from the UK and other European systems showed the same apparent paradox. Diagnostic procedures decreased, the number of new cases reported decreased, but the proportion of advanced forms and cases sent directly to palliative care increased. The conclusion is consistent with ours: it was not surgery that was the weak link, but timely identification.

Our results are in line with internationally reported trends regarding the effects of the pandemic on digestive oncology. Infection control measures (lockdown, social distancing) and the redirection of medical resources to the care of COVID-19 patients have severely disrupted screening programs and elective endoscopic investigations. In Japan, for example, authorities recommended temporarily suspending cancer screening and postponing non-urgent endoscopies in 2020, which, combined with the population’s fear of going to hospitals, led to a 42% decrease in the number of upper digestive endoscopies performed and a drastic reduction (up to 73%) in new diagnoses of gastric cancer [23].

As a result, there was a decrease in the detection of early gastric cancers during that period and a relative increase in the proportion of tumors at advanced stages at the time of detection. Similar trends were reported in other regions; for example, a global multicenter study (145 centers, 50 countries) found a worsening of clinical stages at diagnosis in 2020, with an increase in metastatic cases and a decrease in the number of oncological surgeries performed [24]. Similarly, data collected in the UK showed a 58% reduction in weekly diagnoses of digestive cancer during the initial lockdown, associated with a decrease in endoscopic activity [25,26]. All of this confirms that many patients with gastrointestinal cancer were not diagnosed in time during the pandemic, with their disease continuing to progress in the absence of routine investigations.

In Romania, limited access to medical services during the 2020 state of emergency had similar consequences. A study conducted in a regional center in the west of the country also reported an increase in the proportion of patients presenting in advanced stages (III–IV) immediately after the restrictions were lifted, suggesting that the temporary interruption of diagnostic endoscopies during the pandemic led to the delayed detection of many cases of gastric cancer [27].

Globally, the World Health Organization has reported that over 50% of countries experienced partial or complete disruptions to cancer services in 2020, with an anticipated increase in preventable deaths through prevention and early detection [28,29]. In fact, it is estimated that approximately 2.3 million cancer surgeries were postponed or canceled in the first year of the pandemic [28,30,31,32,33]. In this context, the decrease in survival observed in our pandemic cohort is a predictable result: the stage of the disease at the time of diagnosis remains the most important prognostic factor in gastric cancer, with 5-year survival falling from 70% in localized forms to less than 10% in the presence of systemic metastasis. Our study confirms this reality, with patients diagnosed during COVID-19 having more unfavorable outcomes, mainly attributable to the advanced stage of the tumor at presentation.

In terms of the global context of our statistics, we note that the distribution of stage at diagnosis varies substantially across health systems. In Japan and South Korea, long standing nationwide gastric cancer screening programs and high endoscopy capacity allow most patients to be diagnosed at an early stage, with stage I accounting for up to seventy percent of cases before the pandemic and only modest increases in stage IV disease reported during COVID-19 [18,23,34,35,36,37]. Even after the disruption caused by the pandemic, these countries saw only moderate increases in the percentage of advanced cases; for example, Japan saw an 11% jump in the monthly incidence of stage IV gastric cancer cases in 2020 [26], and in Korea, the proportion of stage IV cases in patients over 40 increased significantly in 2021, but the absolute values remained below those in countries without extensive screening [19]. In contrast, Romania does not have a population-based early detection program for gastric cancer; as a result, even before the pandemic, almost half of patients already presented with metastatic disease. Even before the pandemic, a high proportion of patients in our center presented with advanced or metastatic gastric cancer. This likely reflects structural features of the local healthcare system, including the absence of an organized gastric cancer screening program, limited use of opportunistic endoscopy in asymptomatic individuals and modest public awareness of alarm symptoms. These baseline vulnerabilities probably amplified the impact of the pandemic on stage distribution and survival. The pandemic has exacerbated this situation, raising the proportion to over two-thirds, which is an alarming level [16,38]. This level is close to the upper limit of what has been reported internationally in 2020–2021. For example, data from Western centers showed increases in advanced stages but not necessarily up to two-thirds of cases. The fact that our statistics exceed many international reports suggests that the local healthcare system has been particularly affected, possibly due to more difficult access to medical services during lockdown, patients’ reluctance to go to hospital for fear of infection, and the absence of any preventive endoscopic screening. Even countries without systematic screening, such as the UK or the US, experienced major delays in the diagnosis of gastrointestinal cancers in 2020, a sign that the problem was global; however, the magnitude of the effect in our region was probably exacerbated by pre-existing vulnerabilities in early detection [39,40,41].

Another aspect to discuss is that maintaining curative treatment capacity (surgery, chemotherapy) was not sufficient to avoid a deterioration in prognosis if diagnosis was delayed. At our center, oncological surgery continued almost uninterrupted during the pandemic years. However, the large number of patients arriving at advanced stages led to a net decrease in survival compared to the pre-pandemic period. This observation highlights an important message: the availability of treatment cannot compensate for the lack of early diagnosis. In other words, for cancers such as gastric cancer, timely identification remains essential; surgery performed at an advanced stage may not offer the same chances of survival as surgery performed at an early stage. Consequently, it is imperative that in future health crises, healthcare systems protect the cancer diagnosis chain. Also, in the long term, the implementation of early detection programs could substantially improve the prognosis, reducing the proportion of terminal diagnoses even in the absence of major disruptions to the healthcare system.

Our study has a number of methodological and clinical limitations that should be taken into account. First, its retrospective design and monocentric nature, with all patients coming from a single tertiary center, limit the external validity of our findings and reduce their generalizability to other settings and healthcare systems. These results therefore mainly reflect care pathways and constraints in a Romanian tertiary center and should be extrapolated with caution; future research should rely on larger, multicenter cohorts, ideally at national or regional level, to confirm and extend these observations. Second, several clinically important patient-level variables were not available in a standardized form for the entire cohort. Comorbidities, performance status, symptom onset and individual COVID-19 infection were documented in a heterogeneous way, often only as short free-text notes, so we could not derive a usable Charlson index, ECOG score, symptom-to-diagnosis interval or patient-level COVID-19 status for all included patients. Because of this, we cannot rule out residual confounding by differences in case mix between the pre-pandemic and pandemic cohorts, and the observed period effect should be interpreted as an adjusted association rather than a fully disentangled causal estimate. Moreover, the exclusion of the 44 patients with unknown M stage from stage specific analyses could bias the magnitude of the observed stage shift if these individuals systematically differed from fully staged patients in ways that we could not capture. The proportion of missing M data was modest, which makes a major qualitative distortion of the results less likely, yet the possibility of some degree of under or overestimation of the true period effect cannot be completely ruled out. Thirdly, patients diagnosed towards the end of the study period had a shorter follow-up interval, which may lead to an underestimation of events such as recurrence or death and limits the study’s ability to assess long-term survival in this subgroup. We were also unable to retrieve complete, procedure-level statistics on upper endoscopy volume, waiting times or outpatient clinic schedules for the whole study period, because these data were not stored in a single, analyzable database. For this reason, we could not quantify changes in diagnostic throughput with the same level of detail as larger national or multicenter studies. Last but not least, it is possible that some cases of gastric cancer with delayed diagnosis in the context of the pandemic may not yet have been recorded by the end of 2021 due to prolonged delays in presentation; a similar phenomenon of undiagnosed cancer cases has also been reported at the European level. These limitations do not negate the importance of the observations obtained, but they do call for caution in interpreting the results and highlight the need for further research through larger, preferably multicenter and prospective studies to confirm and extend these conclusions.

This study is relevant because it provides a detailed and comparative analysis of the impact of the COVID-19 pandemic on the presentation and survival of gastric cancer in a Romanian institutional setting and on an extensive cohort, elements that have not been fully explored in previously published research in the national literature. While some previous Romanian studies have addressed to some extent the effects of the pandemic on digestive cancers, they have focused either on changes in hospitalization volume, stage distribution, or post-pandemic cases, without providing rigorous statistical modeling of survival and without controlling for confounding factors. In contrast, the present study includes 419 consecutively diagnosed patients, providing a direct comparison between the pre-pandemic and pandemic periods, with the application of multivariate analyses that highlight the pandemic as an independent determinant of both metastatic presentation and overall mortality. Furthermore, the study provides a differentiated perspective on how surgical treatment capacity was maintained, while late presentations were accentuated, highlighting the role of diagnostic dysfunctions rather than therapeutic limitations. Through the size of the cohorts, methodological clarity, and level of detail in the survival analysis, the study makes a solid and necessary contribution to understanding the systemic impact of the pandemic on digestive oncology in Romania.

## 5. Conclusions

In our center, the COVID-19 pandemic was associated with a pronounced stage migration and reduced survival in gastric cancer, despite comparable demographics and maintained treatment capacity. The pattern of results is compatible with the hypothesis that diagnostic and referral disruptions contributed to these outcomes, although other unmeasured factors may also have played a role. Safeguarding endoscopic and oncologic diagnostic pathways during systemic crises should remain a priority to avoid preventable late-stage presentations and loss of curative opportunities.

In this retrospective cohort of 419 consecutive patients with gastric cancer from a tertiary center in southern Romania, comparing the pre-pandemic (2018–2019) and pandemic (2020–2021) intervals revealed a clear stage migration and a measurable survival penalty associated with COVID-19. Because demographics, referral geography, and access to oncologic treatment remained similar across periods, our multivariable models could isolate the calendar period as a proxy for system-level pressures on diagnosis. During the pandemic, the proportion of metastatic presentations increased, and diagnosis in the pandemic period remained the strongest independent predictor of both M1 disease at entry and higher mortality, even after adjustment for age, sex, residence, and surgery. Taken together, these findings suggest that fragility may have been greater in the diagnostic and referral chain than in treatment capacity, but this interpretation remains tentative and should be regarded as hypothesis-generating.

Our study adds evidence from a Romanian tertiary center to a broader picture already described for colorectal and other digestive cancers, where the pandemic pushed diagnoses towards advanced stages and reduced opportunities for curative treatment. Together, these converging signals suggest that future crisis planning must protect access to gastrointestinal endoscopy, imaging, and specialist evaluation, so that people with potentially curable cancers are not again seen only when their disease has already become disseminated.

## Figures and Tables

**Table 1:** Baseline characteristics of patients diagnosed with gastric cancer before and during the COVID-19 pandemic.

Characteristic	PRE (2018–2019)	PAN (2020–2021)	*p*-Value
Patients (*n*)	243	176	–
Age (mean ± SD)	66.1 ± 12.8	68.0 ± 10.6	0.098 *
Male sex (%)	58.8%	66.5%	0.137 **
Urban residence (%)	69.1%	73.9%	0.345 **
Surgery (%)	55.1%	47.7%	0.161 **

* Student’s *t*-test. ** Chi-square test.

**Table 2:** Distribution of metastatic disease (M1) according to study period.

Period	M0 (Non-Metastatic)	M1 (Metastatic)	Metastatic (%)	Non-Metastatic (%)	*p*-Value
PRE (2018–2019)	123	92	42.8%	57.2%	<0.001 *
PAN (2020–2021)	57	103	64.4%	35.6%	<0.001 *
**Total**	180	195	52%	48%	–

* Chi-square test. Note: denominators are restricted to patients with documented M status; records with missing M were excluded from stage-specific comparisons.

**Table 3:** Cox proportional hazards regression for overall survival.

Variable	Coefficient (β)	Std. Error	*p*-Value	HR	95% CI (Lower–Upper)
**Period (PAN vs. PRE)**	+0.54	0.09	**<0.001**	**1.72**	1.41–2.03
Age (years)	+0.01	0.01	0.17	1.01	0.99–1.03
Male sex	−0.78	0.23	**0.01**	**0.46**	0.29–0.74
Urban residence	+0.07	0.19	0.64	1.07	0.75–1.52
Surgery (yes vs. no)	−0.11	0.20	0.59	0.90	0.62–1.31

Notes: Outcome = overall survival; time scale used in models = OS in days; HR > 1 indicates higher mortality risk; variables entered simultaneously (enter method).

**Table 4:** Multivariate logistic regression for predictors of advanced (M1) disease at diagnosis.

Variable	Coefficient (β)	Std. Error	*p*-Value	OR	95% CI (Lower–Upper)
**Period (PAN vs. PRE)**	+0.97	0.22	**<0.001**	**2.63**	1.68–4.11
Age (years)	+0.01	0.01	0.27	1.01	0.99–1.03
Male sex	−0.12	0.17	0.49	0.89	0.64–1.24
Urban residence	+0.09	0.19	0.63	1.10	0.76–1.61

Notes: Dependent variable = presence of metastases (M1 = 1). Model adjusted for age, sex, and residence. An odds ratio (OR) greater than 1 indicates a higher probability of advanced-stage disease.

## Data Availability

The authors declare that the data of this research are available from the corresponding authors upon reasonable request.

## Abbreviations

The following abbreviations are used in this manuscript:

COVID-19	Coronavirus Disease 2019
OS	Overall Survival
HR	Hazard Ratio
OR	Odds Ratio
CI	Confidence Interval
SD	Standard Deviation
IQR	Interquartile Range
TNM	Tumor–Node–Metastasis classification
M0	Non-metastatic disease
M1	Metastatic disease
PRE	Pre-pandemic period (2018–2019)
PAN	Pandemic period (2020–2021)
